# High-resolution imaging of living mammalian cells bound by nanobeads-connected antibodies in a medium using scanning electron-assisted dielectric microscopy

**DOI:** 10.1038/srep43025

**Published:** 2017-02-23

**Authors:** Tomoko Okada, Toshihiko Ogura

**Affiliations:** 1 Biomedical Research Institute, National Institute of Advanced Industrial Science and Technology (AIST), Central 2, Umezono, Tsukuba, Ibaraki 305-8568, Japan

## Abstract

Nanometre-scale-resolution imaging technologies for liquid-phase specimens are indispensable tools in various scientific fields. In biology, observing untreated living cells in a medium is essential for analysing cellular functions. However, nanoparticles that bind living cells in a medium are hard to detect directly using traditional optical or electron microscopy. Therefore, we previously developed a novel scanning electron-assisted dielectric microscope (SE-ADM) capable of nanoscale observations. This method enables observation of intact cells in aqueous conditions. Here, we use this SE-ADM system to clearly observe antibody-binding nanobeads in liquid-phase. We also report the successful direct detection of streptavidin-conjugated nanobeads binding to untreated cells in a medium via a biotin-conjugated anti-CD44 antibody. Our system is capable of obtaining clear images of cellular organelles and beads on the cells at the same time. The direct observation of living cells with nanoparticles in a medium allowed by our system may contribute the development of carriers for drug delivery systems (DDS).

Nanometre-scale-resolution analytical methods for specimens in liquids are indispensable tools in biology, chemistry and nanotechnology^1^,^2^,^3^,^4^,^5^. In biological fields, direct detection of intact cells and/or bacteria is desirable for analysing the mechanisms behind biological phenomena^6^. Recently, drug delivery systems (DDS) have been widely used to maximise the effect of a medicine whilst minimising its side effects^7^,^8^,^9^. In the development of such systems, significant effort has been devoted to nanotechnological techniques for delivering small-molecular-weight drugs, proteins and genes to desirable target tissues^8^,^10^,^11^. In several cases, the systems use nanometre-sized particles of emulsions, polymers, silica and liposomes^9^,^12^,^13^. Because nanoparticles are more easily incorporated into cells than microparticles, DDS using nanometre-sized particles offers an advantage^7^. On the contrary, nanoparticles in water are hard to detect using traditional optical or electron microscopy. The resolution of a traditional optical microscope is limited to 200 nm because of the diffraction limit of light. Recently, super-resolution fluorescence microscopes have been developed with resolutions of approximately 20 nm^3^,^14^; however, observations with these methods require specimens to be fluorescently labelled^3^,^14^. The spatial resolution of a conventional scanning electron microscope (SEM) is approximately 3 nm; however, using conventional SEM, observing wet biological specimens or nanoparticles in water is difficult because the specimen chamber is in a high-vacuum condition^15^. Atmospheric holders have been developed since the 1970 s to allow such observations^2^,^4^,^16^,^17^,^18^. These traditional atmospheric holders receive radiation damage and the system is difficult to obtain clear contrast of the unstained biological specimens^17^. Recently, environmental SEM has been developed, which enables observing of the wet samples under vapour pressure condition^19^,^20^,^21^. Further, recently high-resolution scanning transmission electron microscopy (TEM) successfully observed fully hydrated living cells without staining^22^,^23^. This system enabled clear contrast detection of the living yeast in limited radiation damage at 30 nm resolution^22^.

In a recent study, we developed a novel imaging technology named as scanning electron-assisted dielectric microscopy (SE-ADM), which enables observation of intact cells, bacteria and protein particles in water with very low radiation damage and high-contrast imaging without staining or fixation^24^,^25^,^26^,^27^. The spatial resolution of the SE-ADM system reached 8 nm^26^. Moreover, our system is capable of producing high-contrast images of untreated biological specimens in aqueous conditions^26^,^27^. Biological samples are enclosed in a liquid holder composed of tungsten (W)-coated silicon nitride (SiN) film and are not directly exposed to electron beam. Irradiated electrons are almost absorbed in a tungsten layer on the SiN thin film; thus, the negative electric-field potential arises at this position^24^. This negative potential is detected at the bottom measurement terminal through the specimen in water. The detection mechanism is based on the difference of electric dipoles of the water and specimen materials^24^. Because water has a high electric permittivity; the electric-potential induced by the irradiated electron in W-coated SiN film is propagated to the lower SiN film through the sample solution^24^. On the other hand, as the biological specimens consist of organic materials (for example amino acids and lipids) with low electric permittivity, they decrease the transmission electric signal^24^,^25^,^26^,^27^. Therefore, our system enables high-contrast imaging with low radiation damage.

In the previous report, we firstly showed our SE-ADM system observing the untreated living mammalian cells under aqueous condition^27^. In contrast, here, we first report that the SE-ADM system is capable of observing antibody-binding nanoparticles in liquid-phase. Moreover, we successfully observe nanobeads directly binding to mammalian cancer cells via antibodies in a medium and their intracellular structure at the same time.

## Results

**Figure 1:**
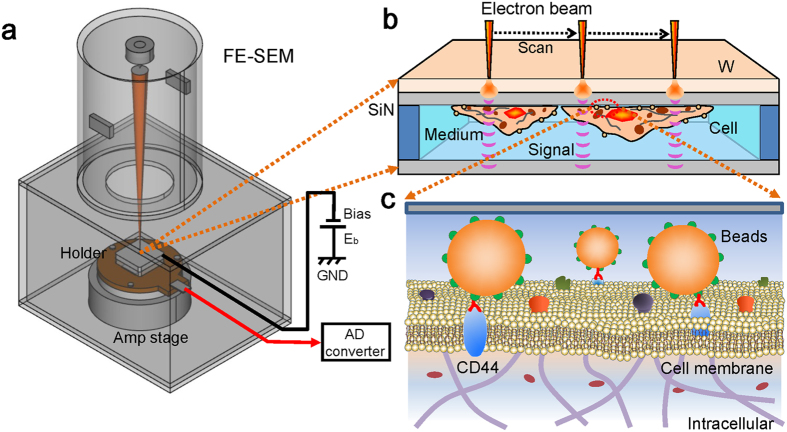
Overview of our dielectric microscopy of the SE-ADM system using a culture-dish holder. (**a**) A schematic diagram of the SE-ADM system based on FE-SEM. The liquid-sample holder with nanoparticles and/or cultured cells is mounted on the pre-amplifier-attached stage, which is introduced into the specimen chamber. The scanning electron beam is applied to the W-coated SiN film at a low acceleration voltage. The measurement terminal under the holder detects the electrical signal through liquid specimens. (**b**) Overview of the liquid holder in the cultured cancer cells bound with 100-nm beads. After 4–5 days of culturing in the dish holder, the cancer cells stained with streptavidin-conjugated 100-nm beads and biotin-conjugated anti-CD44 antibodies were sealed in the bottom sample holder. The cancer cells were attached to the upper SiN film, and its W-coated side was irradiated by the scanning electron beam. (**c**) A conceptual diagram of the cell membrane with streptavidin-conjugated 100-nm polystyrene beads and biotin-conjugated anti-CD44 antibodies via streptavidin-biotin interaction. Biotin is not shown in the diagram.

Figure 1 shows a schematic outline of the SE-ADM system for detecting culture cells with antibody-binding nanoparticles. Our SE-ADM system is based on a field-emission scanning electron microscope (FE-SEM) (Fig. 1a). Mouse cancer cells (4T1E/M3)^28^,^29^,^30^ are cultured in the dish holder containing medium^27^. The holder, which contains cells, is separated from the plastic culture dish and attached to an acrylic holder^27^. Cultured cancer cells in the interspace between SiN films are maintained in medium conditions under atmospheric pressure (Fig. 1b). The binding of the nanobeads onto the cells via antibodies is directly observed by our SE-ADM system under medium conditions (Fig. 1c).

**Figure 2:**
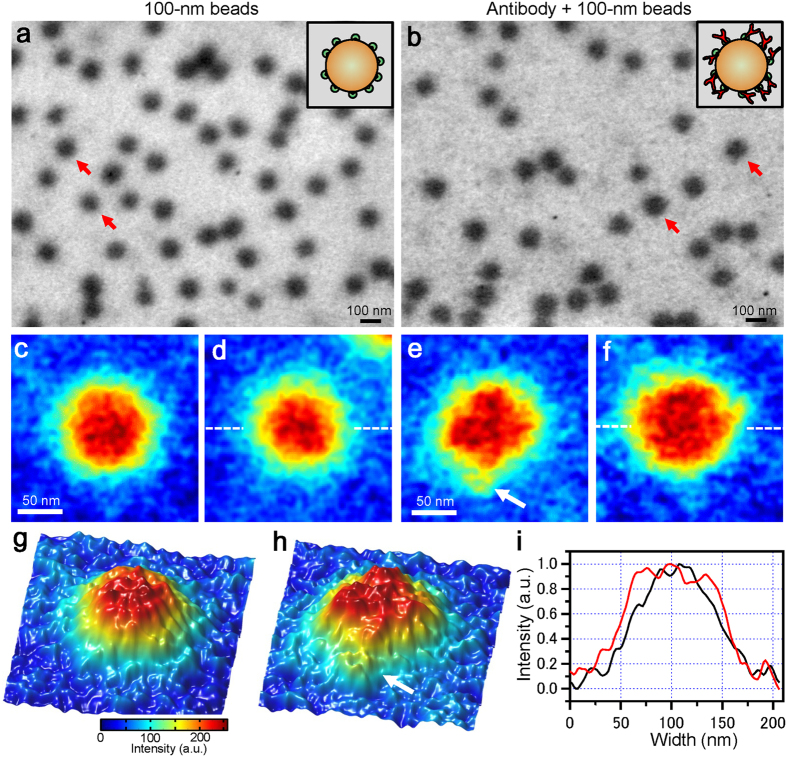
Imaging of polymeric beads in liquid using the SE-ADM system. (**a**) A dielectric image of the 100-nm polystyrene beads conjugated with streptavidin in liquid at 50,000× magnification under a 3.6-kV electron beam acceleration. The image was filtered using 2D GF (11 × 11 pixels, 1.2σ) after background subtraction. Several clear black spheres dispersed over the whole area represent the 100-nm polystyrene beads. A schematic figure in the upper-right square shows polystyrene beads conjugated with streptavidin on their surface. (**b**) A dielectric image of 100-nm polystyrene beads conjugated with streptavidin bound to biotin-conjugated anti-CD44 antibodies in liquid at 50,000× magnification. The image of antibody-binding beads shows a rough surface with a small projection form. A schematic figure in the upper-right square shows polystyrene beads conjugated with streptavidin and antibodies on their surface. (**c**,**d**) Expanded pseudo-colour images of 100-nm beads indicated by the red arrows in (**a**). These beads surfaces are smooth. (**e**,**f**) Expanded pseudo-colour images of 100-nm beads to which anti-CD44 antibodies are bound (indicated by the red arrows in (**b**)). These beads have very rough surfaces. (**g**) A 3D colour map of the same beads in (**c**). (**h**) A 3D colour map of the same beads to which antibodies are bound in (**e**). The white arrow suggests the anti-CD44 antibody. (**i**) Comparison of the line plots of bead centres. The black line shows the 100-nm beads of (**d**), while the red line shows the beads to which antibodies are bound in (**f**). The range of the red line is wider than that of the black line. Scale bars are 100 nm in (**a**) and (**b**), and 50 nm in (**c**) and (**e**).

Initially, we observe the streptavidin conjugated polystyrene beads under untreated liquid conditions (Fig. 2a). These beads are clearly shown to have spherical form and to be dispersed in water at 50,000× magnification. The beads’ diameter is detected to be approximately 100 nm in its SE-ADM image (Fig. 2a). Then, we observe the mixed solution of the streptavidin-conjugated 100-nm polystyrene beads and biotin-conjugated anti-CD44 antibody (Fig. 2b). The biotin-conjugated antibodies are bound by the streptavidin-conjugated beads. The image of the antibody-binding beads shows a rough surface with a small spinal form (Fig. 2b). The 100-nm polystyrene beads indicated by red arrows in Fig. 2a and b are magnified and shown in a pseudo-colour map for detailed analysis (Fig. 2c–f). The surfaces of spherical beads without antibodies look rather smooth (Fig. 2c,d); in contrast, the beads bound to antibodies exhibit many spines on their surfaces (Fig. 2e,f). We compare the surface structures of Fig. 2c and e using three-dimensional (3D) pseudo-colour maps (Fig. 2g,h), which clearly show their differences. The white arrow in Fig. 2e and h exhibits an antibody-like structure; moreover, line plots of both beads’ centres (Fig. 2d,f) clearly show that the antibody-binding beads are significantly wider in diameter than those without antibodies (Fig. 2i).

**Figure 3:**
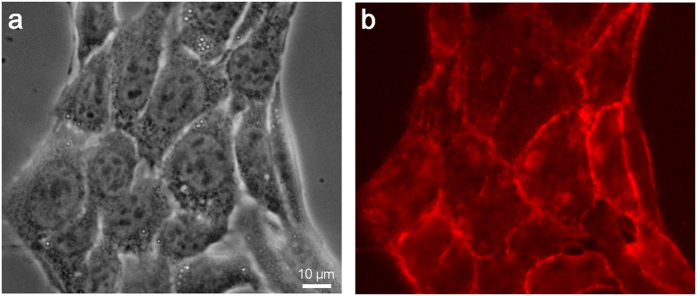
Optical phase-contrast and fluorescence observation images of cells stained with biotin-conjugated anti-CD44 antibodies and streptavidin-conjugated rhodamine. (**a**) Optical phase-contrast image of antibody-stained cultured cells obtained with an optical microscope at 400× magnification. (**b**) Fluorescence image of anti-CD44 immunostained cells obtained from an optical microscope with a fluorescence filter at 400× magnification. Anti-CD44 antibodies are localized on the cell membranes. The scale bar represents 10 μm in (**a**).

Next, we directly observe the binding of nanobeads to cancer cells via anti-CD44 antibodies in a medium condition. CD44 is known as a hyaluronic acid (HA) receptor and a prominent marker of malignancy in several types of cancers^31^,^32^,^33^,^34^. Therefore, an understanding of the binding mechanism of CD44 to cancer cells may aid in the design of effective DDS therapeutic techniques for cancer patients^35^. 4T1E/M3 cells are stained first with the biotin-conjugated anti-CD44 antibodies and then with streptavidin-conjugated rhodamine, then observed via optical fluorescent microscopy (Fig. 3). Phase-contrast (Fig. 3a) and fluorescence (Fig. 3b) images clearly show that the CD44 protein is widely localized on cellular membranes.

**Figure 4:**
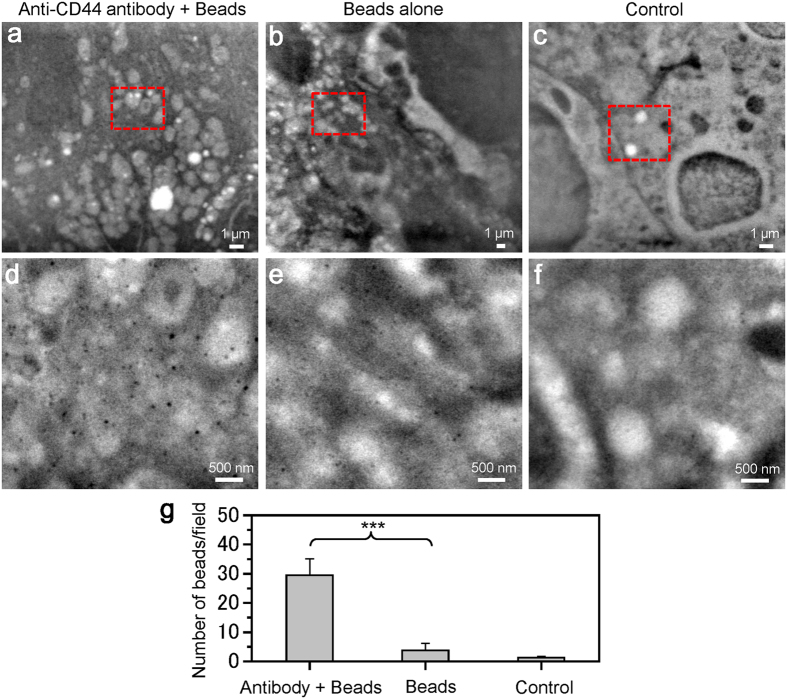
SE-ADM images of nanobeads binding to cancer cells via anti-CD44 antibodies. (**a**) An image of the streptavidin-conjugated 100-nm beads binding to cells via biotin-conjugated anti-CD44 antibodies in medium using the SE-ADM system at an electron beam-acceleration of 6 kV, 5,000× magnification and −32 V bias. (**b**) An image of the streptavidin-conjugated 100-nm beads binding cells without anti-CD44 antibodies using SE-ADM system at an electron beam-acceleration of 10 kV and 3,000× magnification. (**c**) An image of unstained cancer cells at electron beam-acceleration of 8 kV and 5,000× magnification. (**d**–**f**) Expanded images of the red boxes in (**a**–**c**) with 20,000× magnification. In (**d**), many clear black spherical particles are dispersed over the whole area. In (**e**), few spherical beads are observed. In (**f**), almost no spherical beads are observed. (**g**) The average number of nano-beads/field with or without anti-CD44 antibody to the 4T1E/M3 cells. The average number of nano-beads/field is 29.5 with antibodies, 3.75 without antibodies and 1.25 in the control, in four scanned images of each condition at 20,000× magnification (an image size of 5.8 μm × 4.8 μm). Values are means ± SD; ***p < 0.0001. The scale bars represent 1 μm in (**a**–**c**), 500 nm in (**d**–**f**).

We now directly observe the streptavidin-conjugated nanobeads bound to the biotin-conjugated anti-CD44 antibody on the cell surface using the SE-ADM system. Mouse cancer cells (4T1E/M3) are incubated with biotin-conjugated anti-CD44 antibody for 30 min; then, they are stained with 100-nm streptavidin-conjugated beads. Through this treatment, the 100-nm beads conjugated with anti-CD44 antibodies are bound to the CD44 protein on the cell membrane. A low-magnification image of the cells taken by the SE-ADM system clearly shows the intracellular structure (Fig. 4a–c). To detect the 100-nm beads, we image the central region of the cell at a magnification of 20,000× (Fig. 4d–f). The black spherical particles are found to be dispersed on the cell membranes when they are bound to anti-CD44 antibodies (Fig. 4d), whereas few beads are detected on the cells stained by 100-nm beads alone (Fig. 4e) and almost no beads are detected on the untreated cells (Fig. 4f). The average number of 100-nm beads/field is 29.5 with anti-CD44 antibodies, 3.75 without antibodies and 1.25 in the control, in four scanned images at each condition of 20,000× (Fig. 4g).

**Figure 5:**
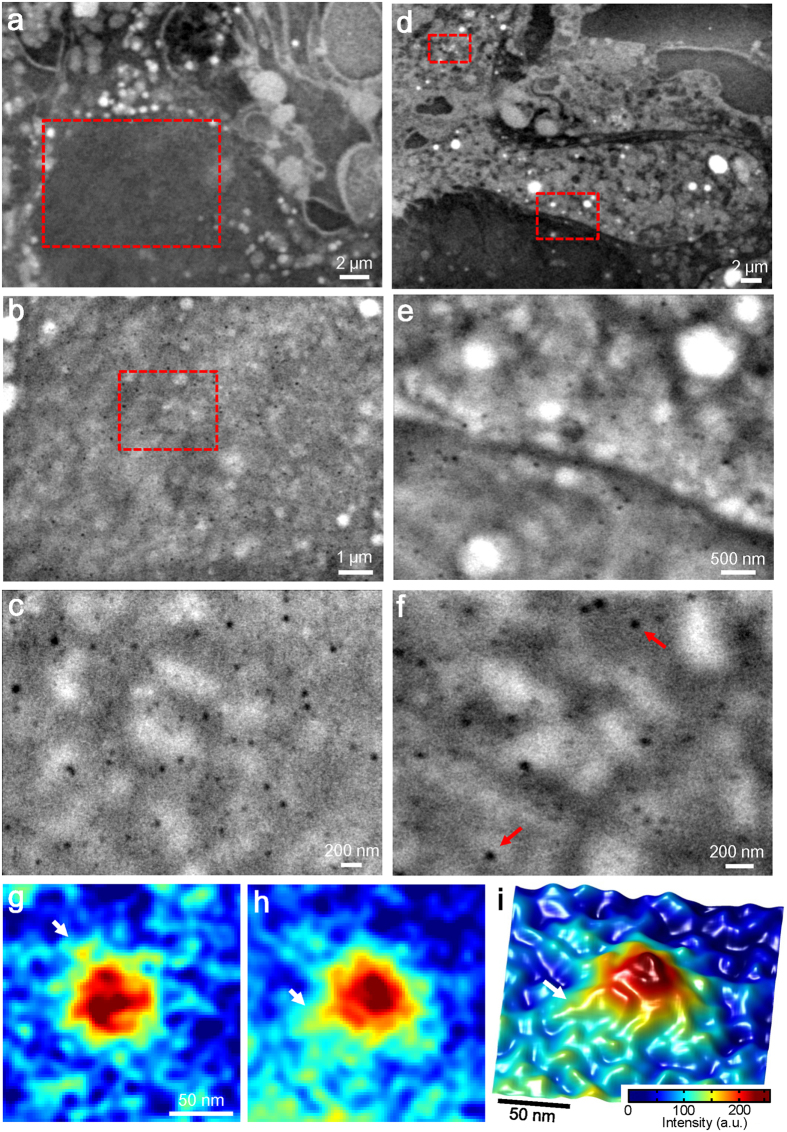
High-resolution images of anti-CD44 antibodies binding 100-nm beads on the membranes of cancer cells. (**a**) A dielectric image of the nucleus region of a cancer cell stained with antibody-binding 100-nm beads at an electron beam acceleration of 6 kV, 3,000× magnifications and −32 V bias. (**b**) An expanded image of the red boxed area in (**a**) at 10,000× magnification. (**c**) High-resolution image of the red boxed area in (**b**) at 30,000× magnification. Clear black spherical particles are dispersed over the whole area. (**d**) Another image of the cancer cells stained with antibody-binding 100-nm beads at an electron beam acceleration of 6 kV, 3,000× magnification and −32 V bias. (**e**) An expanded image of the red boxed area at the bottom centre in (**d**) at 20,000× magnification. (**f**) High-resolution image of the red boxed area in (**d**) at the top left at 40,000× magnification. This area also shows 100-nm beads of clear black spheres. (**g**,**h**) Pseudo-colour maps of enlarged images of the bead binding areas indicated by red arrows in (**f**). (**i**) 3D colour map of (**h**). The white arrows suggest the anti-CD44 antibody. The scale bars are 2 μm in (**a**) and (**d**), 1 μm in (**b**), 500 nm in (**e**), 200 nm in (**c**) and (**f**), 50 nm in (**g**) and (**i**).

The CD44-binding 100-nm beads on the living cells are further analysed at various cell positions and high magnification (Fig. 5). Figure 5a shows a 3,000× magnification of the SE-ADM image of the nuclear region of the cell. The large spherical black object at the bottom of Fig. 5a is a typical mammalian nucleus. For detailed observation, the centre of the nucleus region (red square) is scanned at 10,000× magnification (Fig. 5b); many small black dots are detected on cell membrane above the nucleus. This suggests that these dots correspond to the 100-nm beads bound to CD44 proteins, which can be clearly observed at a magnification of 30,000× (Fig. 5c). Similar spherical beads are shown in other cell regions (Fig. 5d–f). Figure 5d shows another living cell imaged by SE-ADM system at 3,000× magnification, which shows clear intracellular structures. Figure 5d shows the border area of the nucleus and cytoplasm; a section of this image (red square at the bottom) is shown at 20,000× magnification in Fig. 5e. Another cytoplasmic region in Fig. 5d (the red square at the top left) is shown at 40,000× magnification in Fig. 5f. Both images (Fig. 5c and f) clearly show many 100-nm beads dispersed over the whole area of the cell membrane. Figure 5g and h show colour maps of enlarged images of the 100-nm beads indicated by the red arrows in Fig. 5f. Figure 5i shows a 3D colour map of Fig. 5h. The white arrows in Fig. 5g–i indicate protrusions from the bead’s surface, which are suspected to be the anti-CD44 antibody.

## Discussion

Recently, the super-resolution fluorescence microscopies reached to the resolution which is higher than 50 nm^3^,^14^. However, this method needs to use the fluorescence dye or fluorescence beads. On the other hand, our SE-ADM system enables to observe the beads and/or specimens without fluorescence dye. Moreover, the spatial resolution of our SE-ADM system reached 8 nm measured by 25–75% rising edge of IgM protein particle^26^. High-resolution scanning TEM and cryo-TEM enabled observation of the high-contrast imaging of the biological specimens in water and/or in ice^6^,^22^. Our results presented here demonstrate that our SE-ADM system (Fig. 1) clearly observed 100-nm polystyrene beads bound to antibodies in a liquid phase (Fig. 2b), without staining or metal coating. The density of polystyrene beads is 1.03 g/ml, which is very close to the liquid density. Therefore, observing the bead’s structure with very clear contrast using a traditional liquid-sample holder for SEM is difficult because the irradiated electron beam is scattered and absorbed by both the liquid and polystyrene beads with antibodies. Our system enabled clear detection of the antibody-binding beads, which showed wider diameters than those without antibodies (Fig. 2c–i). Using our SE-ADM technique, we previously reported the direct observation of intact mammalian cancer cells and changes of their intracellular structure under medium conditions^27^. In DDS, polymeric nanoparticles are commonly used as the drug carriers^9^. Therefore, our system would be useful for analysing the mechanism by which drugs are delivered by observing drug carrier particles binding to the living cells in medium.

CD44 is a complex transmembrane glycoprotein initially identified as a receptor for HA and a lymphocyte-homing receptor^31^,^32^,^34^, which is involved in many processes, including cellular adhesion, angiogenesis, inflammation and tumour development^32^,^34^. Much evidence suggests that CD44 is a prominent marker of several types of cancer-cell malignancy, including invasion and metastasis^34^, and may be an important target for DDSs^35^. CD44 protein exhibits high expression on the cell membranes of mouse cancer 4T1E/M3 cells (Fig. 3). In this study, we successfully detected streptavidin-conjugated 100-nm nanobeads bound to the CD44 protein on the cell membrane via a biotin-conjugated anti-CD44 antibody (Figs 4 and 5). These SE-ADM images showed several 100-nm beads dispersed over the entire cell area (Figs 4d and 5c,f). Notably, our system could simultaneously observe 100-nm beads and intracellular structure without metal staining (Fig. 5a–f). Conventional super-resolution microscopes, including optical microscope^3^,^14^ and X-ray microscopes^36^,^37^ are difficult to detect both the undamaged structures of polystyrene beads and intracellular structure without staining in the medium condition^4^,^21^. By contrast, our system can clearly observe both structures without metal staining or fixation. Future application of our SE-ADM system to the DDS or correlative work with lipid stain to show specificity would contribute the establishment of useful DDS.

At present, the spatial resolution of our SE-ADM system remains unsatisfactory for more detailed structural analysis of membrane proteins bound by nanobeads. For a more precise analysis of such protein structures, the spatial resolution may be better than 3 nm. To approach this, we are currently constructing an SE-ADM system based on a SiN film thinner than 10 nm and a fine electron beam of approximately 1-nm diameter using super high-resolution FE-SEM.

## Conclusion

In conclusion, we have reported the successful direct observation of non-fluorescence 100-nm polystyrene beads binding to antibodies in aqueous condition by our SE-ADM system. Moreover, we have performed the clear detection of streptavidin-conjugated nanobeads binding to untreated cell membranes in a liquid medium via biotin-conjugated antibodies using our system. These cells were placed between two SiN films in a liquid holder and detected using our newly developed SE-ADM system. Our system was capable of simultaneously imaging both structures of the cellular organelles and antibody-binding 100-nm polystyrene beads. Therefore, our SE-ADM system would contribute the analysis of the mechanism by which drugs are delivered to cells. Our method can also be applied to various liquid samples across a broad range of scientific fields, including nanotubes, organic materials and ceramics.

## Methods

### 100-nm-beads and CD44-antibody preparation

Polystyrene-matrix particles having 100-nm diameter conjugated with streptavidin in PBS (phosphate buffered saline) solution were obtained from Micromod Partikeltechnologie GmbH (Rostock, Germany). The beads density was 1.03 g/ml. The 100-nm beads alone in liquid solution (2 μl) were added and sealed in the liquid-sample holder as a control sample.

Biotin-conjugated rat anti-mouse-CD44 antibodies (catalog #: 553132) were obtained from BD Bioscience. The biotin anti-CD44 antibodies (1 μl) were mixed with the streptavidin-conjugated 100-nm beads (1 μl) and attached to the surfaces of the beads via biotin-streptavidin interaction. Then, this mixture solution was introduced to the liquid-sample holder.

### 4T1E/M3 cell culture and sample preparation

Mouse breast cancer cells (4T1E/M3) were established as previously described^28^,^29^,^30^. Cells were cultured in a high-glucose RPMI-1640 medium containing 10% fetal calf serum (FCS) and 20 mM HEPES at 37 °C under 5% CO_2._ After adding the culture medium (described above; 1.5 ml/dish) to the culture dish attached under the SiN-Al holder, cells (4 × 10^4^; 20 μl /dish) were seeded and cultured at 37 °C under 5% CO_2_. The medium was changed after 2–3 days, and the cells formed a sub-confluent or complete confluent monolayer on the SiN membrane in the holder after 4–5 days.

### Immunolabelling

The cells seeded in the dish holder were stained with biotin-conjugated anti-mouse-CD44 antibodies (BD Bioscience, 1/50, 50 μl) diluted by the 1:1 mixture of PBS and medium for 30 min at 4 °C, washed twice with the mixture solution and stained with streptavidin-conjugated polystyrene particles of 100-nm diameter (Micromod Partikeltechnologie GmbH, 1/30, 50 μl) for 30 min at 4 °C. After washing twice, the holder was observed by the SE-ADM system.

### Tungsten deposition on the upper SiN film

A 50-nm-thick SiN film supported by a 0.4 × 0.4 mm window in a Si frame (4 × 4 mm, 0.38-mm-thick; Silson Ltd., Northampton, UK) was coated with tungsten using a magnetron sputtering device (Model MSP-30T, Vacuum Device Inc., Japan), as previously described^24^.

### Liquid-sample holder and culture-dish holder

The liquid-sample holder was formed as previously described^24^. Briefly, the liquid-sample holder comprised an upper Al holder and lower acrylic resin portion that maintained the sample solution at atmospheric pressure between the SiN films^27^. The upper W-coated SiN film was attached to the Al holder with double-sided tape, and the W-layer on SiN film was connected to the Al holder by silver conductive ink. A hand-made Al holder with a Si frame was attached under a 35-mm culture dish square hole in the centre by double-sided tape, as previously described^27^. The culture-dish holder was subsequently UV sterilised for 17–18 h.

4T1E/M3 mouse breast cancer cells were cultured in the holder dish and stained with biotin-conjugated anti-mouse-CD44 antibodies and streptavidin-conjugated 100-nm polystyrene beads as described above. Next, the Al holder containing cells was separated from the plastic culture dish, attached upside down to another SiN film on an acrylic plate and sealed^27^. The Al holder received a voltage bias of approximately −32 V (Fig. 1a).

### High-resolution SE-ADM system and FE-SEM setup

The FE-SEM (JSM-7000F, JEOL, Tokyo, Japan)-based high-resolution SE-ADM imaging system is shown in Fig. 1a. The liquid-sample holder was mounted onto the SEM stage, and the detector terminal was connected to a pre-amplifier under the holder^26^,^27^. The electrical signal from the pre-amplifier was fed into the AD converter after low-pass filtering, as has been previously described^27^. The LPF and electron beam-scan signals were logged by a PC through an AD converter at a sampling frequency of 50 kHz. SEM images (1,280 × 1,020 pixels) were captured at 3,000–50,000× magnification with a scanning time of 80 s, working distance of 7 mm, EB acceleration voltage of 3.6–10 kV and current of 10 pA.

### Optical-phase microscope and fluorescence image

Cultured 4T1E/M3 cells in a 35-mm-diameter glass-bottomed dish (Matsunami Glass Ind., Ltd., Osaka, Japan) were stained with biotin-conjugated anti-mouse CD44 antibodies (BD Bioscience, 1/100) for 30 min at 4 °C, washed twice with PBS and stained with streptavidin-conjugated Rhodamine (Vector Laboratories, Inc., 1/100) for 30 min at 4 °C. After washed twice with PBS, cells were visualised at 400× magnification using an optical phase microscope (AXIO Observer A1; Carl Zeiss, Oberkochen, Germany). Fluorescent images of the cancer cells were observed using a fluorescence filter of excitation/emission wavelength 565/620 nm.

### Image processing

SE-ADM signal data from the AD converter were transferred to a personal computer (Intel Core i7, 2.8 GHz, Windows 7), and high-resolution SE-ADM images were processed from the LPF signal and scanning signal using the image-processing toolbox of MATLAB R2007b (Math Works Inc., Natick, MA, USA). Original SE-ADM images were filtered using a two-dimensional (2D) Gaussian filter (GF) with a kernel size of 7 × 7 pixels and a radius of 1.2 σ. Background subtraction was achieved by subtracting SE-ADM images from the filtered images using a broad GF (400 × 400 pixels, 200 σ).

### Statistical analysis

Differences in number of nano-beads with antibody and without antibody conditions were analysed using one-way ANOVA followed by Bonferroni’s multiple comparisons test. GraphPad Prism (Version 4; GraphPad Softwere, San Diego, CA USA) was used for statistical analysis.

## Additional Information

**How to cite this article:** Okada, T. and Ogura, T. High-resolution imaging of living mammalian cells bound by nanobeads-connected antibodies in a medium using scanning electron-assisted dielectric microscopy. *Sci. Rep.* **7**, 43025; doi: 10.1038/srep43025 (2017).

**Publisher's note:** Springer Nature remains neutral with regard to jurisdictional claims in published maps and institutional affiliations.
